# Visual feedback improves bimanual force control performances at planning and execution levels

**DOI:** 10.1038/s41598-021-00721-9

**Published:** 2021-10-27

**Authors:** Hyun Joon Kim, Joon Ho Lee, Nyeonju Kang, James H. Cauraugh

**Affiliations:** 1 Department of Human Movement Science, Incheon National University, Incheon, South Korea; 2 Division of Sport Science, Sport Science Institute, and Health Promotion Center, Incheon National University, Incheon, South Korea; 3 Neuromechanical Rehabilitation Research Laboratory, Incheon National University, 119 Academy-ro, Yeonsu-gu, Incheon, South Korea; 4 Department of Applied Physiology and Kinesiology, University of Florida, Gainesville, USA

**Keywords:** Neuroscience, Psychology

## Abstract

The purpose of this study was to determine the effect of different visual conditions and targeted force levels on bilateral motor synergies and bimanual force control performances. Fourteen healthy young participants performed bimanual isometric force control tasks by extending their wrists and fingers under two visual feedback conditions (i.e., vision and no-vision) and three targeted force levels (i.e., 5%, 25%, and 50% of maximum voluntary contraction: MVC). To estimate bilateral motor synergies across multiple trials, we calculated the proportion of good variability relative to bad variability using an uncontrolled manifold analysis. To assess bimanual force control performances within a trial, we used the accuracy, variability, and regularity of total forces produced by two hands. Further, analysis included correlation coefficients between forces from the left and right hands. In addition, we examined the correlations between altered bilateral motor synergies and force control performances from no-vision to vision conditions for each targeted force level. Importantly, our findings revealed that the presence of visual feedback increased bilateral motor synergies across multiple trials significantly with a reduction of bad variability as well as improved bimanual force control performances within a trial based on higher force accuracy, lower force variability, less force regularity, and decreased correlation coefficients between hands. Further, we found two significant correlations in (a) increased bilateral motor synergy versus higher force accuracy at 5% of MVC and (b) increased bilateral motor synergy versus lower force variability at 50% of MVC. Together, these results suggested that visual feedback effectively improved both synergetic coordination behaviors across multiple trials and stability of task performance within a trial across various submaximal force levels.

## Introduction

Bimanual force control is frequently involved in conducting various activities of daily living^1^. Moreover, successful coordination between forces produced by two hands contributes to accomplishing the goals of bimanual movements^2^. Conventional perspectives on bimanual force coordination assume that force outputs from each hand need to be coupled such as a single unit so that these coupling patterns improve bimanual force control performance^3^. However, recent studies raised a proposition that two hands may consistently interact and produce forces in a synergetic way to improve bimanual force performances. The reason being that the central nervous system (CNS) tends to select the synergetic movements in numerous options denoting the motor abundance (i.e., the CNS benefits from the plenty of degrees of freedom in motor actions) rather than attempting to find a specific method meaning the motor redundancy (i.e., the CNS confronts a problem in choosing excessive degrees of freedom in motor actions)^4,5^.

The uncontrolled manifold (UCM) analysis is frequently used for estimating individual’s motor synergetic patterns between two limbs^4,6^. A classical perspective on errors in human movement by Schmidt and Lee suggested two types of motor errors: (a) errors in motor planning and (b) errors in motor execution. Motor planning errors appear across multiple trials because of inappropriate selection of a new motor action plan, whereas motor execution errors occur within a trial because of inaccurate simultaneous motor corrections^7^. Thus, a within-trial analysis can determine how the variability in the degrees of freedom is modulated over time during the execution of a single trial, and a between-trials analysis can reveal how the performers select or plan a new motor action across trials potentially requiring a higher level of cognitive functions^8,9^. Given that the UCM findings mainly focus on changes in motor control patterns across multiple trials, this approach provides an elegant way to explore movement variability and coordinative behaviors in the motor system at the planning levels^7,8^.

During bimanual force control tasks, the UCM approach can define fundamental elements as pairs of left- and right-hand forces (i.e., mean forces for each trial) collected from multiple trials. Two variability components of the fundamental elements across multiple trials involve (a) good variability denoting the variance of fundamental elements projected to the UCM line and (b) bad variability indicating the variance of fundamental elements projected to the orthogonal (ORT) line. Despite no effects of good variability on task performance, increased amount of good variability indicates greater flexibility in selecting motor solutions (i.e., various combinations of left and right forces equal to the targeted level). However, altered bad variability influences task performance so that a greater amount of bad variability across multiple trials may interfere with the stability of overall task performance indicating improved bimanual force control performances (e.g., higher task error values averaging across trials). Importantly, the index of bilateral motor synergies is a proportion of good variability to bad variability. Following three cases in greater bilateral motor synergies indicate either more flexible or stable coordinative behaviors across multiple trials: (a) increased good variability without changes in bad variability (more flexibility and no changes in stability of overall task performances), (b) decreased bad variability without changes in good variability (more stability of overall task performance and no changes in flexibility), and (c) increased good variability and decreased bad variability (more flexibility and stability of overall task performances). Prior studies suggested that greater bilateral motor synergies patterns with a reduction of bad variability may contribute to improvements in bimanual force control performances within a trial indicating more stability of overall task performances^6,10,11^.

Several UCM studies examined changes in bilateral motor synergies across multiple trials during bimanual force control tasks according to specific constraints (i.e., organism, task, and environment constraints) that may influence an individual’s coordination functions^10,12,13^. For example, findings from studies on elderly people and individuals post-stroke revealed less bilateral motor synergies with higher task error and variability within a trial as compared with age-matched controls because of their potential organism constraints such as impaired sensorimotor processing^11,13,14^. Moreover, the asymmetrical task goals during bimanual force control increased bilateral motor synergies with more precise force control in healthy young adults in comparison to the symmetrical force production tasks^6,10,12^. Although these findings indicated that certain constraints may simultaneously influence levels of bilateral motor synergies across multiple trials and force control performances within a trial, whether specific changes in motor synergic patterns across multiple trials are related to an altered stability of task performances within a trial is still unclear.

To determine potential relationship between bilateral motor synergies and bimanual force control performances, we modulated visual information during bimanual force control tasks. Previous findings reported that the presence of visual information improved force control performances, as indicated by force accuracy, variability, and regularity with better force coordination function (e.g., more negative correlation between two hands) within a trial, whereas the absence of visual information impaired the force control capabilities and facilitated more positive correlation patterns^15,16^. Further, some studies reported that the modulation of visual information strongly affected individual’s motor control strategies using higher cognitive functions as indicated by altered motor synergic patterns across multiple trials^17,18^.

Thus, the purpose was to determine the effect of two visual conditions and three targeted force levels on bilateral motor synergies and force control performances during isometric force control tasks in healthy young adults. We administered bimanual isometric force control tasks by extending their wrists and fingers under two visual feedback conditions (i.e., vision and no-vision) and three targeted force levels (i.e., 5%, 25%, and 50% of maximum voluntary contraction: MVC) because of potential different motor control strategies across trials and within a trial influenced by different task difficulty^19–21^. For each targeted force level, we additionally computed potential correlations between changes in bilateral motor synergies and stability of overall task performance from vision to no-vision conditions. Given that the positive effects of visual feedback on motor control capabilities^22^, we hypothesized that the presence of visual feedback would increase bilateral motor synergies and enhance bimanual force control performances, and further the increase in bilateral motor synergies would be related to more improvements in bimanual force control performances.

## Results

### UCM findings: bilateral motor synergies, good variability, and bad variability

**Figure 1:**
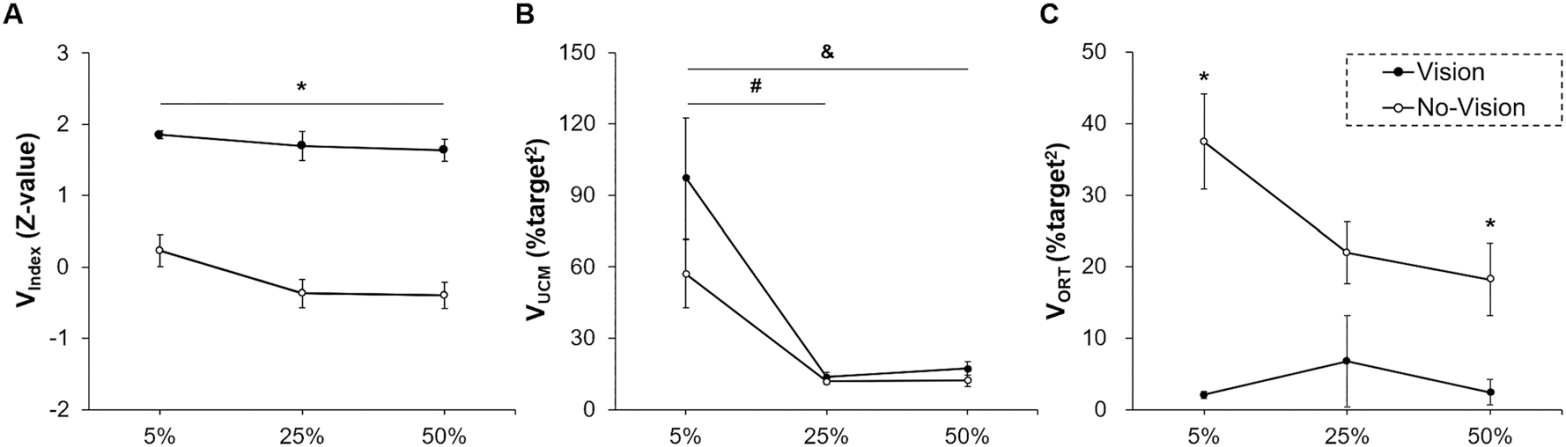
Bimanual coordination across multiple trials using UCM approach (*M* ± *SE*). (**A**) bilateral motor synergies (V_Index_) showing a significant Visual Condition main effect. (**B**) Good variability (V_UCM_) showing a significant Force Level main effect. (**C**) Bad variability (V_ORT_) showing a significant Visual Condition × Force Level interaction. Asterisk (*) denotes a significant difference between visual conditions. Number sign (#) indicates a significant difference between 5 and 25% of MVC. Ampersand (&) means a significant difference between 5 and 50% of MVC.

Repeated measures ANOVA on the Visual Condition × Force Level (2 × 3) design for the V_Index_ showed a significant Vision Condition main effect [*F* (1, 13) = 226.476; *P* < 0.001; partial η^2^ = 0.946; Fig. 1A]. Specifically, the values of V_Index_ were significantly greater in the vision condition than those in the no-vision condition. Two-way repeated measures ANOVA on the V_UCM_ demonstrated a significant Force Level main effect [*F* (1.042, 13.549) = 19.045; *P* = 0.001; partial η^2^ = 0.594]. As shown in Fig. 1B, the post-hoc analysis on the Force Level main effect findings showed higher values of the V_UCM_ at 5% of MVC than those at 25% of MVC (*P* = 0.002) and 50% of MVC (*P* = 0.002). However, despite a tendency that V_UCM_ in the vision condition (*M* ± *SE* = 42.701 ± 8.354) was relatively greater than those in the no-vision condition (*M* ± *SE* = 27.008 ± 4.686), the analysis failed to show a significant Visual Condition main effect [*F* (1, 13) = 2.932; *P* = 0.111; partial η^2^ = 0.184].

The analysis on the V_ORT_ revealed a significant Vision × Force level interaction [*F* (2, 26) = 4.078; *P* = 0.029; partial η^2^ = 0.239; Fig. 1C]. The post-hoc analyses indicated that the values of V_ORT_ were significantly less in the vision condition than those in the no-vision condition at 5% of MVC (*P* < 0.001) and 50% of MVC (*P* < 0.001). At 25% of MVC, the analysis similarly showed different trends in the V_ORT_ between vision and no-vision conditions (*P* = 0.051). These findings indicate that interlimb coordination across multiple trials as indicated by bilateral motor synergies was improved when visual feedback was available, and further the presence of visual feedback reduced bad variability components in bilateral motor synergies. These patterns were observed across all targeted force levels.

### Bimanual force control performances: force accuracy, variability, regularity, and correlation coefficients

**Figure 2:**
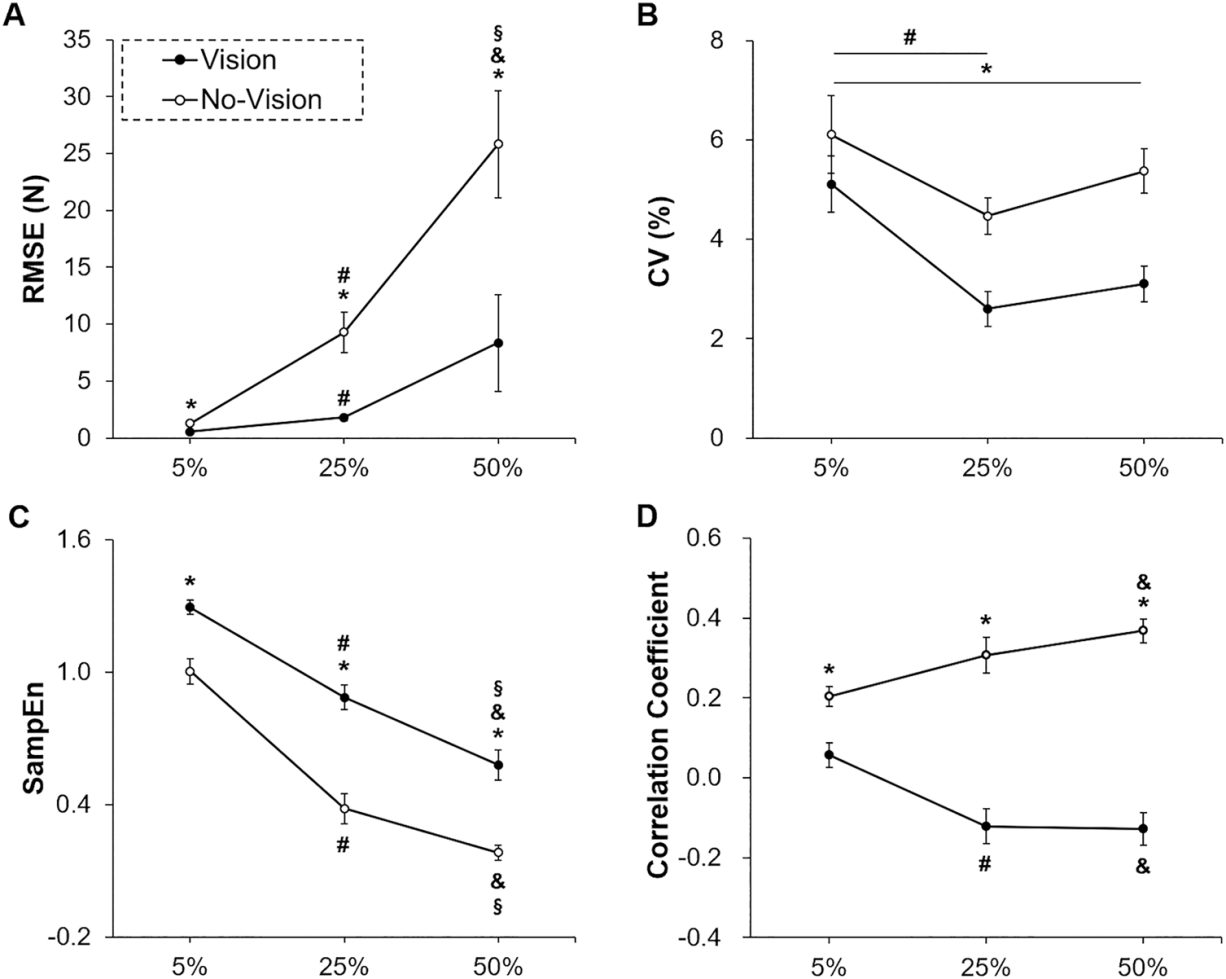
Bimanual force control performance within a trial (*M* ± *SE*). (**A**) Force accuracy (RMSE) showing a significant Visual Condition × Force Level interaction. (**B**) Force variability (CV) showing significant Visual Condition and Force Level main effects. (**C**) Force regularity (SampEn) showing a significant Visual Condition × Force Level interaction. (**D**) Correlation coefficients between hands showing a significant Visual Condition × Force Level interaction. Asterisk (*) denotes a significant difference between visual conditions. Number sign (#) indicates a significant difference between 5 and 25% of MVC. Ampersand (&) means a significant difference between 5 and 50% of MVC. Section sign (§) shows a significant difference between 25 and 50% of MVC*.*

Repeated measures ANOVA on the two-way Visual Condition × Force Level (2 × 3) for the RMSE revealed a significant Vision × Force level interaction [*F* (1.328, 17.261) = 27.193; *P* < 0.001; partial η^2^ = 0.677; Fig. 2A]. The post-hoc analyses indicated that the values of RMSE were significantly less in the vision condition than those in the no-vision condition for each targeted force level (*P* < 0.001). Further, in the no-vision condition, the values of RMSE significantly increased as the force level elevated (*P* < 0.001).

A two-way repeated ANOVA on the CV showed two significant main effects: (a) Vision Condition [*F* (1, 13) = 61.273; *P* < 0.001; partial η^2^ = 0.825; Fig. 2B] and (b) Force Level [*F* (1.157, 15.045) = 5.594; *P* = 0.028; partial η^2^ = 0.301]. The Vision Condition main effect findings revealed less CV in the vision condition than those in the no-vision condition. In addition, post-hoc analysis on the Force Level main effect showed higher CV at the 5% of MVC than those at the 25% of MVC.

Analysis of the SampEn revealed a significant Vision × Force Level interaction [*F* (2, 26) = 5.162; *P* = 0.013; partial η^2^ = 0.284; Fig. 2C]. The follow-up tests showed higher SampEn in the vision condition than those in the no-vision conditions across the three targeted force levels (*P* < 0.001). Further, for both visual conditions, SampEn decreased as the targeted force level increased (*P* < 0.001).

The analysis on the correlation coefficients between hands revealed a significant Vision × Force level interaction [*F* (2, 26) = 19.268; *P* < 0.001; partial η^2^ = 0.597; Fig. 2D]. The post-hoc analyses indicated that the values of correlation coefficients between hands in the vision condition were significantly lower than those in the no-vision condition for each targeted force level (*P* < 0.01). In the vision condition, the values of correlation coefficients between hands at 25% and 50% of MVC significantly decreased in comparison to those at 5% of MVC (*P* < 0.01). In the no-vision condition, the values of correlation coefficients between hands at 50% of MVC were significantly higher than those at 5% of MVC (*P* = 0.002). Taken together, these findings indicate that the presence of visual feedback efficiently improved bimanual force control performances within a trial, as indicated by less force error, variability, regularity, and correlation coefficients between hands regardless of different targeted force levels.

### Correlation findings: bilateral synergies versus force control performance

**Figure 3:**
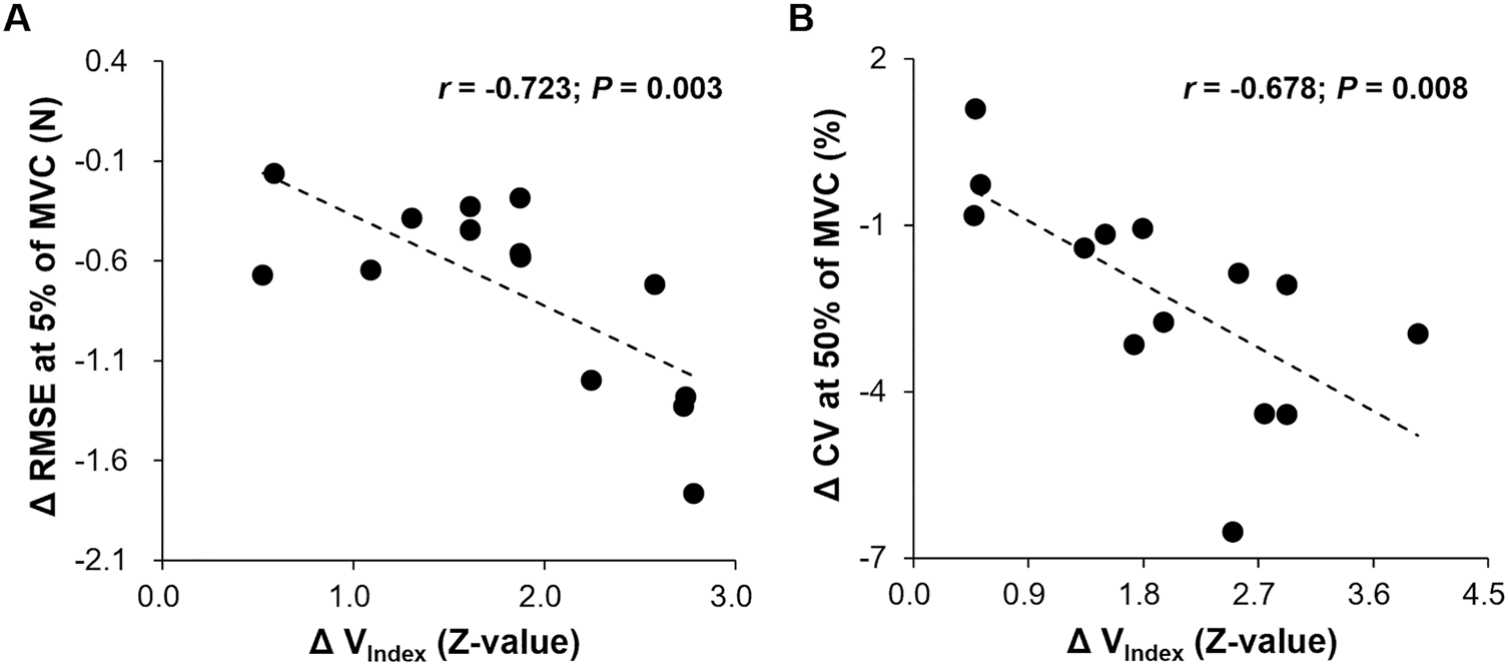
Correlation findings between changes in bilateral motor synergies and bimanual force control performances from vision to no-vision conditions. (**A**) Negative correlation between bilateral motor synergy (V_Index_) and force accuracy (RMSE) at 5% of MVC. (**B**) Negative correlation between bilateral motor synergy (V_Index_) and force variability (CV) at 50% of MVC.

To determine whether changes in bilateral motor synergies across multiple trials were related to changes in bimanual force control performances (i.e., force accuracy, variability, regularity, and correlation coefficients between hands) within a trial from the no-vision to vision conditions, we performed Pearson’s correlation analyses for each targeted force level. The correlation findings showed that increased values of the V_Index_ from the no-vision to vision conditions were significantly related to a reduction in the RMSE at 5% of MVC (Fig. 3A). At 50% of MVC, increased values of the V_Index_ from the no-vision to vision conditions were significantly related to decreased CV (Fig. 3B). These findings indicate that advanced interlimb coordination patterns across multiple trials were potentially related to greater improvements in bimanual force control performances within a trial at 5% and 50% of MVC.

## Discussions

The purpose was to determine the effect of two visual conditions and three targeted force levels on bilateral motor synergies and force control performances during isometric force control tasks in healthy young adults. Moreover, we examined the correlation between changes in bilateral motor synergies and force control performances to specify whether enhanced bimanual coordinative actions across multiple trials were related to increased stability of task performance within a trial. The combined findings revealed that in the vision condition the performers significantly increased bilateral motor synergies while reducing bad variability across multiple trials. Moreover, the presence of visual feedback significantly improved bimanual force control performances within a single trial, as indicated by higher force accuracy, less force variability, lower force regularity and decreased correlation coefficients between hands. Importantly, we observed significant correlations between an increase in bilateral motor synergy and improvements in force control performances when the visual feedback was provided.

The UCM findings revealed that the values of bilateral motor synergies between two hands significantly increased with a reduction of bad variability components for each targeted force level in the vision condition. According to Newell’s constraints theory^23,24^, cooperative bimanual movement patterns in the motor system were primarily dependent on three constraints within a single trial: (a) organism constraints (e.g., a performer’s capability), (b) environmental constraints (e.g., extrinsic feedback), and (c) task constraints (e.g., task difficulty). In addition to the within-trial findings, previous UCM studies demonstrated the effects of three constraints on interlimb coordination patterns across multiple trials, as they observed decreased bilateral motor synergies in a specific population (e.g., older adults)^13,14,25^ and easier task difficulty (e.g., symmetrical tasks)^10,12^. Similarly, the current findings confirmed the influence of an environmental constraint (i.e., visual information) on bilateral motor synergies consistent with prior results^12,13^. Moreover, the two targeted force levels below 50% of MVC did not alter bilateral motor synergies although the ratio of good and bad variability was affected by the task constraint^11,26^. These findings indicated that the performers showed a tendency to maintain the level of bilateral motor synergies during various submaximal force modulations by interactively modulating the ratios of good variability and bad variability across multiple trials.

The presence of online visual feedback increased bilateral motor synergies implicating more cooperative motor actions between two hands with the reduction of bad variability components across multiple trials. Freitas et al. proposed the stability-optimality trade-off phenomenon in human behaviors^27^. Specifically, an optimality strategy postulates that the central nervous system selects an optimal motor solution from numerous motor elements by minimizing the variance of force elements produced by two limbs along the UCM line. On the other hand, a stability strategy indicates the organization of numerous motor elements for stabilizing task performance by decreasing the variance of force elements along the ORT line resulting in greater index of bilateral motor synergies (i.e., higher good variability relative to bad variability). These two motor control strategies may independently influence actions generating synergetic interlimb movements^5,27–29^. Taken together, our findings suggest that when the simultaneous task-related visual feedback is available, the performer may prefer the stability strategy for successful task performances across multiple trials via increasing bilateral motor synergies while minimizing bad variability components.

As we expected, bimanual force control performances within a trial were more improved in the vision condition, as indicated by decreased task error, variability, regularity, and correlation coefficients between hands. These findings indicated that visual feedback increased the stability of overall task performances during bimanual force control. Presumably, providing visual feedback facilitates activation of visuomotor networks contributing to online motor corrections and task stabilization during isometric force control^12,15,30,31^. In addition, the availability of online visual information may positively regulate firing rates on neuromuscular systems by decreasing the neural noise of synaptic input and motor neuron pools^12,32^. Moreover, the performers increased force irregularity in the vision condition indicating more compensatory and adaptive force outputs between hands. A prior study reported increased values of approximate entropy (ApEn) on bimanual forces with the presence of visual information^15^. Given that SampEn analysis may have higher independence of results according to different data lengths^33^, we additionally confirmed force regularity reduced by visual information with the advanced nonlinear approach. Perhaps, the presence of visual information decreases environmental entropy while simultaneously increasing motor entropy, referred to as compensatory trade-off effects^34,35^. Finally, the greater reduction of values in correlation coefficients between hands with visual feedback indicated that visual feedback improved bimanual force coordination function within a trial consistent with previous findings^15,36^. These findings support that more negative correlation patterns implicating more compensatory and less stable anti-phase cooperative actions between hands contributed to a reduction of task error during bimanual force control tasks^15^. Indeed, the effects of visual feedback on altered bimanual coordination patterns across multiple trials and within a trial suggested that the motor system can effectively modulate and adapt bimanual feedback motor control strategies^37^.

Importantly, our correlation findings revealed that an increase in bilateral motor synergy across multiple trials with visual feedback was positively correlated with the improvements in force control performances (i.e., a reduction of task error and force variability) at 5% and 50% of MVC, respectively. Ranganathan and Newell reported that the presence of visual information during bimanual finger force control tasks contributed to reducing disturbances on the variability in degrees of freedom at the execution level and facilitating the use of numerous motor solutions at the planning level^8^. Further, the findings suggested that synthesizing motor control processes between execution and planning levels may consequently increase the accuracy and efficiency of motor outputs from the motor system^8^. As revealed in a neuroimaging study^38^, force control improvements in the vision condition were significantly related to visuomotor processing with greater parietal cortical activations. Given that the parietal cortex was highly involved in both motor execution and planning^38–40^, the presence of online visual information may increase neural resources for the visuomotor integration contributing to more adaptive and corrective motor strategies at the execution level and updating motor control strategies at the planning level. Accordingly, the current findings tentatively support a possibility of the relation between bilateral synergetic coordination strategies at the planning level (i.e., between multiple trials) and traditional motor variability of degrees of freedom produced by two hands at the execution level (i.e., within a single trial).

Despite potential effects of visual feedback on bilateral motor synergies and force control performances, these findings were cautiously interpreted. First, we found a significant relationship between an increase in bilateral motor synergies across multiple trials and more improvements in bimanual force control performances within a trial at limited targeted force levels (5% and 50% of MVC). Previous force control findings showed that force variability (i.e., CV) changed according to U-shape^41–43^ and force regularity was altered according to an inverted U-shape^44,45^ below 50% of MVC. Presumably, given more improved force control capabilities at 25% of MVC as compared with 5% and 50% of MVC, performers may not require specific motor control strategies stabilizing task performances via increasing motor synergetic coordination. Moreover, previous studies evidenced that force control performances within a trial were dependent on the amount of visual feedback (e.g., decreased RMSE and CV with greater visual angle and spatial amplitude of visual feedback) with the facilitation of visuomotor networks such as parietal cortex, premotor cortex, and supplementary motor cortex)^30,46^. Thus, future studies should investigate potential brain activation patterns (e.g., functional connectivity within visuomotor areas) underlying altered the bilateral motor synergies and force control performances between vision and no-vision conditions. Presumably, these findings would provide more information regarding motor system’s interactive control strategies at the execution and planning levels.

## Conclusions

The current study revealed that visual feedback during bimanual isometric force control tasks significantly increased bilateral motor synergies across multiple trials while simultaneously reducing bad variability components. Further, improvements in bimanual force control performances within a trial were identified in the vision condition as indicated by lower force error, variability, regularity, and correlation coefficients between hands. Importantly, we observed that a higher frequency of bilateral motor synergetic patterns was positively related with more improvements in force control performances between vision and no-vision conditions. These results suggested that task-related visual feedback effectively improved both synergetic coordination behaviors across multiple trials and stability of task performance within a trial across various submaximal force levels. Despite the positive effects of task-related visual information on bimanual force control across multiple trials and within a trial, how altering visual feedback influences bimanual force control strategies is still unknown. Thus, future studies should investigate the effects of variations on visual feedback (e.g., visual gain or frequency) on bilateral motor synergies and force control performances.

## Methods

### Participants

Fourteen young adults (mean ± standard deviation age = 21.6 ± 2.3 years; eight females and six males) participated in this study. All participants were right-handed healthy individuals without musculoskeletal deficits in their upper extremities (self-reported) and cognitive dysfunctions (Mini-Mental State Exam score > 26)^47^. To calculate appropriate sample size^48^, we performed a priori power analysis based on the pilot data using G*Power software (version 3.1.9.7), and confirmed that 14 participants in a within-subjects design were minimally required (power = 0.99 and alpha = 0.05). The current study protocols were approved by the University of Florida’s Institutional Review Board, and we confirmed that all methods were performed in accordance with the relevant guidelines and regulations. All participants read and signed an informed consent before starting the test.

### Experimental setup

**Figure 4:**
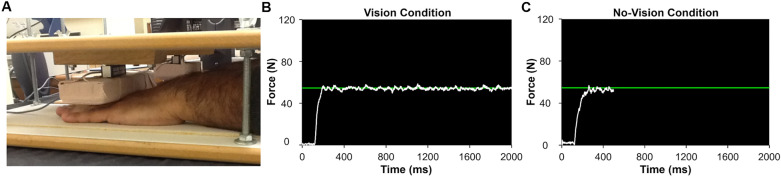
Experimental setup. (**A**) Bimanual force control tasks with wrist and fingers extension. (**B**) Visual feedback condition. (**C**) No-visual feedback condition.

We used isometric force control paradigms during bimanual wrists and fingers extension movements consistent with the prior experimental designs^11,13,49^. Initially, the participants sat 78 cm away from a 43.2 cm LED monitor (1024 × 768 pixels; refresh rate = 100 Hz) and placed their forearms on the desk in comfortable positions (i.e., 15–20° of shoulder flexion and 20–40° of elbow flexion). Next, the participants placed their hands and fingers under two separate customized platforms embedded with a force transducer for each hand (MLP-75, Transducer Techniques, 4.16 × 1.27 × 1.90 cm, range = 75 lbs., 0.1% sensitivity). During the tasks, the participants extended their hands and fingers upward against the platforms to produce isometric forces (Fig. 4A).

Initially, the participants bimanually conducted three maximal voluntary contraction (MVC) trials (a duration of MVC trial = 6 s and an inter-trial interval for the rest = 60 s). Using the mean values of three peak force outputs as the sum of forces generated by both hands across the MVC trials, we calculated three submaximal targeted force levels (i.e., 5%, 25%, and 50% of MVC) for each individual. These submaximal force levels represent a wide range of forces generated in many activities of daily living^15,50,51^, and further prior findings showed that people produced different force control strategies depending on the altered targeted force levels^11,21,26,49^.

The goal of each submaximal isometric force control trial was to match and maintain bimanual force production (i.e., the sum of forces produced by two hands) around a targeted force level for 20 s. Based on previous studies^31,43,49^, we administered two different visual conditions: (a) vision (Fig. 4B) and (b) no-vision (Fig. 4C). Specifically, in the vision condition, the LED monitor simultaneously displayed the performer’s summed forces generated by both hands with a white line trajectory and a targeted force level, a green line centered on the monitor during each 20 s trial. In the no-vision condition, we removed the white force trajectory after the first 5 s, and then the performer saw only a targeted force level for the remaining 15 s. During the task, we instructed all participants to focus on the visual feedback displayed on the LED monitor to minimize any potential effects of direct vision on their hands. Further, given that the participants situated their hands and fingers under two separate customized platforms, this experimental circumstance prevented that direct vision on the hands augmented overall visual information. Participants completed 12 consecutive trials for each experimental block (i.e., 6 experimental blocks = 2 visual conditions × 3 targeted force levels), for a total 72 submaximal force control trials. During these submaximal force control tasks, we randomly assigned the six experimental blocks to participants with each block. Further, controlling potential effects of the altered amount of visual feedback across various targeted force levels on force control performances is crucial. To maintain the same visual feedback across each experimental block, we used a constant visual angle (= 1°) reflecting both visual distance and gain variables consistent with previous suggestion^52^.

A custom LabVIEW Program (National Instruments, Austin, USA) conducted standardized testing procedures and data collection. All force signals were sampled at the rate of 100 Hz using a 16-bit analog-to-digital converter (A/D; NI cDAQ-9172 + NI 9215 and minimal force unit detection = 0.0016 N) and amplified using a 15LT Grass Technologies Physio-data Amplifier System (Astro-Med Inc.) with an excitation voltage of 10 V and a gain of 200.

### Data analyses

For the following offline-data analyses, we initially filtered the raw force data using a bidirectional fourth-order Butterworth filter at 20 Hz of cut off frequency using a custom Matlab program (Math Works™ Inc., Natick, USA). The middle 10 s (i.e., 5–15 s; 1000 data points) of force signals was most important for minimizing transient effects of early or later motor corrections in a trial.

**Figure 5:**
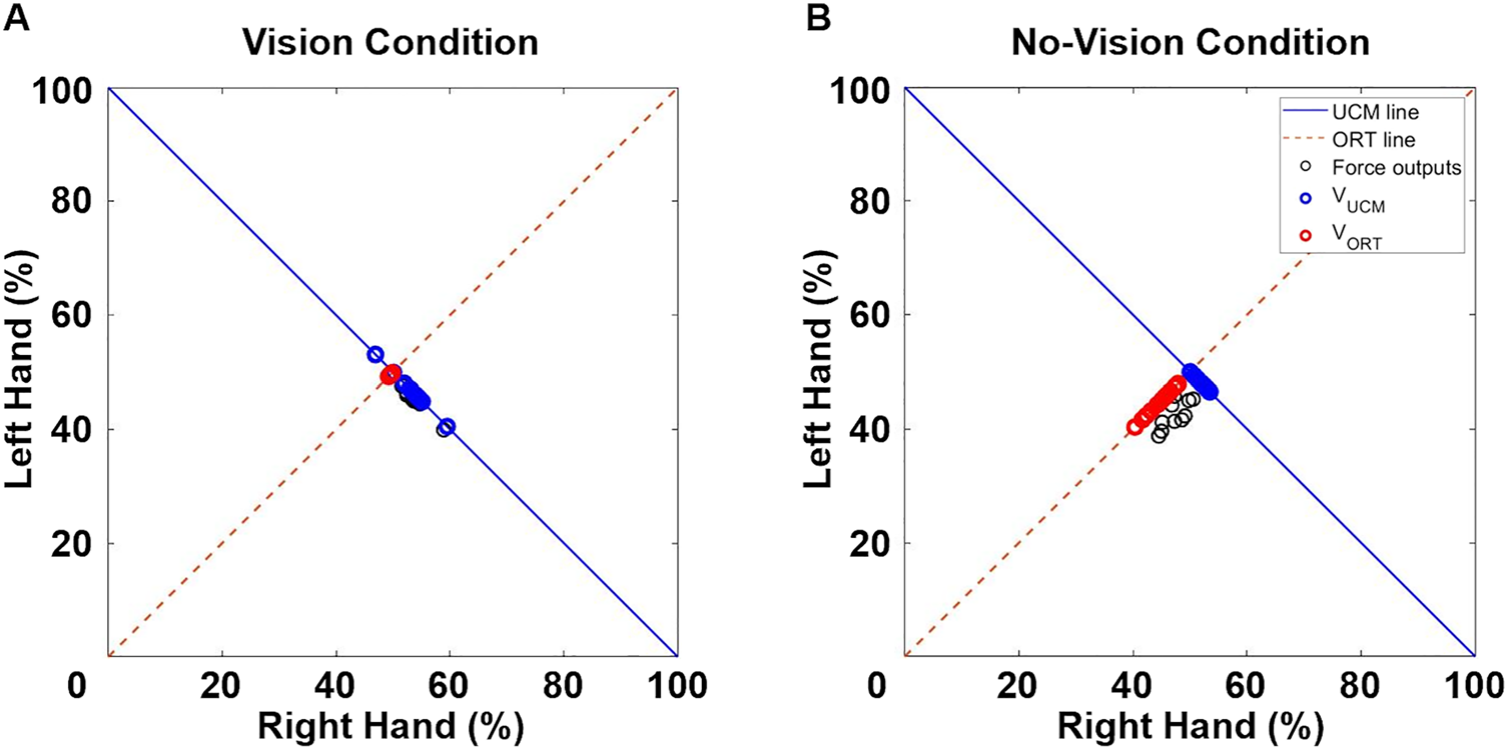
Representative UCM data quantifying variances of fundemental elements projected to the UCM and ORT lines. (**A**) The 12 pairs of nomalized fundamental elements extracted from each trial (i.e., black circles) projected to both UCM line (i.e., blue line) and ORT line (i.e., dotted red line) at 25% of MVC condition in the vision condition and (**B**) No-vision condition.

To estimate bimanual force coordination across trials, we quantified bilateral motor synergies based on the UCM theory^4,6,10,11^. First, we calculated a mean force value for each hand within a trial and normalized the mean force value into fundamental elements pair using a targeted force level. For instance, when a targeted force level is 80 N, a performer may produce 70 N of mean force produced by two hands (i.e., 30 N from left hand and 40 N from right hand). Thus, a pair of normalized fundamental elements included: (a) left hand: (30 N/80 N) × 100 = 37.5% and (b) right hand: (40 N/80 N) × 100 = 50%. Finally, this calculation was repeated across 12 trials for each experimental block, and the 12 pairs of normalized fundamental elements were projected to two distinctive lines: (a) UCM line (Fig. 5A) and (b) ORT line (Fig. 5B), respectively.

The variance components projected to the UCM line are associated with successful motor control capabilities (good variability: V_UCM_). On the other hand, the variance components projected to the ORT line interfere with motor control (bad variability: V_ORT_)^5^. The V_Index_ (i.e., a proportion of V_UCM_ relative to V_ORT_) indicates an index of bilateral motor synergies ranging from − 2 to 2 values (Formula 1). Note that higher values of V_Index_, close to 2, represent better bimanual coordination patterns across multiple trials^13^.

1 $${V }_{Index}=\frac{{V}_{UCM}/{df}_{UCM}-{V}_{ORT}/{df}_{ORT}}{{V}_{TOT}/{df}_{TOT}},$$

where *df*_*UCM*_ shows degrees of freedom of V_UCM_ (df = 1), *df*_*ORT*_ is degree of freedom of *V*_*ORT*_ (df = 1), *V*_*TOT*_ indicates the sum of *V*_*UCM*_ and *V*_*ORT*_, and *df*_*TOT*_ is degrees of freedom of *V*_*TOT*_ (df = 2).

For additional parametric statistical analyses, we performed Z-transformation on all V_Index_ values using Formula (2) consistent with prior findings^10,11^.

2 $${V }_{Index} \left(Z-transformed\right)=0.5 \times \mathrm{ ln }\frac{2+{V}_{Index}}{2-{V}_{Index}}.$$

To assess bimanual force control performance within a trial, we calculated three outcome measures on the sum of forces produced by both hands: (a) force accuracy: root mean square error (RMSE) = , (b) force variability: coefficient of variation (%CV) = SD of force/mean force × 100, and (c) force regularity: sample entropy (SampEn; Formula 3)^33,53^.

3 $$RMSE= \sqrt{\frac{{\sum }_{i=1}^{N}{\left({x}_{i}-T\right)}^{2}}{N},}$$

where ${x}_{i}$ is observed force, *T* is a targeted force level, and *N* is data length.

4 $$\mathrm{SampEn}\left(x, m, r, N\right)=\mathrm{ln }\left[\frac{{C}_{m}\left(r\right)}{{C}_{m+1}\left(r\right)}\right],$$

where $m$ is specific pattern length, $r$ is a criterion of similarity in the time series, and ${C}_{m}\left(r\right)$ indicates occurrence of repetitive patterns of length $m$ in time series $x$ (i.e., force data in the time samples) excluding the self-match^53^. Consistent with a previous study, we used a value of 2 for $m$ and $r$ = 0.2 × standard deviation (SD) of force data^33^.

Finally, to assess bimanual coordination within a trial, we calculated Pearson’s correlation coefficients between left and right forces in time domain^15,54^. In bimanual force control model, more positive values of correlation coefficients (e.g., 0 < *r* ≤ 1) is related to less complicated forces between hands (e.g., in-phase) and impaired force control performance, whereas more negative values of correlation coefficients (e.g., − 1 ≤ *r* < 0) denote more complicated forces between hands (e.g., anti-phase) and improved force control performance^15,49,54^.

### Statistical analyses

All outcome measures (i.e., V_Index_, V_UCM_, V_ORT_, RMSE, CV, SampEn, and correlation coefficients between hands) were analyzed with two-way repeated measure ANOVAs (Visual Condition × Force Level; 2 × 3). We confirmed the normality of all dependent variables across vision and force level conditions using the Shapiro–Wilk’s W test^55^ and Levene’s test^56^. When the sphericity assumption was violated, we reported the degrees of freedom adjustments using Greenhouse-Geisser^57^. For conducting post hoc analysis, we used Bonferroni’s pairwise comparisons. Finally, Pearson’s correlation analysis was used to identify potential relationships between changes in bilateral motor synergy and changes in force control performances including force accuracy, variability, regularity, and correlation coefficients between hands from vision to no-vision conditions (i.e., change in variables = values in vision condition minus values in no-vision condition) for each targeted force level, respectively. All statistical analyses were performed using the IBM SPSS Statistics 22 (SPSS Inc., Chicago, IL, USA) and we set an alpha level at 0.05.

## Data Availability

All data generated or analysed during this study are included in this published article.
